# Combined Measure of Hand Grip Strength and Body Mass Index for Predicting Excess Body Fat in a University Population in Kentucky, USA

**DOI:** 10.3390/diagnostics16081210

**Published:** 2026-04-17

**Authors:** Jason W. Marion, Michael C. Shenkel, Laurie J. Larkin, Jim M. Larkin

**Affiliations:** 1 Master of Public Health Program, Department of Environmental, Public Health, Administration and Medical Sciences, College of Health Sciences, Eastern Kentucky University, Richmond, KY 40475, USA; jason.marion@eku.edu (J.W.M.); michael.shenkel@mymail.eku.edu (M.C.S.); laurie.larkin@eku.edu (L.J.L.); 2 Department of Parks, Recreation, Exercise and Sport Science, College of Health Sciences, Eastern Kentucky University, Richmond, KY 40475, USA

**Keywords:** body fat percentage, bioelectrical impedance analysis, body mass index, BMI, handgrip strength, obesity, adiposity, functional measurement, muscle function, adiposity

## Abstract

**Background/Objectives:** Measures of excess body fat are often more informative for predicting health risk than body mass index (BMI) alone. With obesity prevalence increasing among young adults, this study evaluated whether adding dominant handgrip strength improves prediction of body fat percentage (BF%) and BF%-defined obesity in a university population. **Methods:** Cross-sectional data from 895 students (401 women, 494 men; mean age 19.9 years; fall 2015–spring 2016) in Kentucky, USA were analyzed. BMI was calculated from self-reported height and weight. BF% was estimated using bioelectrical impedance analysis (BIA), and dominant handgrip strength was measured with a hydraulic hand grip dynamometer. Sex-specific linear and logistic regression models assessed associations among BMI, grip strength, relative grip strength, and BF%. **Results:** BMI was a strong predictor of BF% in linear models (R^2^ = 0.74 in women; 0.68 in men). Grip strength alone was not associated with BF% but showed an inverse association when combined with BMI. For BF%-defined obesity, BMI remained the most influential predictor, with grip strength contributing additional predictive value. Among men, age significantly modified these relationships, with marked differences between those aged 18–19 years versus older participants. **Conclusions:** BMI strongly predicted BF% and BF%-based obesity in this cross-sectional study of a predominantly white young adult population. Incorporating handgrip strength modestly improved classification, particularly among women, suggesting that a functional measure like hand grip strength may enhance obesity screening and health communication in young adults.

## 1. Introduction

The prevalence of overweight and obesity among all age groups in the US has risen considerably since 1990. Among older adolescent (ages 15 to 24) and adult (25 years and older) women, the prevalence was 10.1% and 22.8% in 1990, and has risen to 28.8% and 45.6% in 2021, respectively [1]. To characterize prevalence, the Global Burden of Disease 2021 US Obesity Forecasting Collaborators calculated body mass index (BMI) using 134 unique data sources with anthropometric measures. For adults, a BMI of 25–29.9 kg/m^2^ is defined as overweight, and ≥30 kg/m^2^ as obese. Although BMI is widely used, particularly in population-based studies, it has been described as an inadequate measure for research aimed at understanding obesity causes and evaluating interventions [2]. While BMI has been useful as a surrogate for body fat mass, research over the past 25 years has highlighted important limitations of BMI-based approaches for informing public health [3].

The seminal BMI study published in 1972 introduced the term “body mass index” [4] and aimed to develop simple height- and weight-based indices to estimate body fatness [4,5]. BMI, comparable to the Quetelet Index developed in 1823 [6], performed best among simple ratios in correlating with body fatness in a study of 7424 healthy men, most of European ancestry, from five countries [5]. However, Keys and colleagues did not intend BMI to replace methods measuring body density, noting it was not optimal for estimating body fat mass [4,5,7,8].

Although BMI was not intended for clinical determination of excess body fat, by the mid-1990s obesity prevalence had risen sharply and was described as an epidemic in the US [9,10] and a pandemic by 1997 [11]. As evidence linking obesity to adverse outcomes such as cardiovascular disease [12,13] and type-2 diabetes [14] accumulated, public health agencies sought practical tools for obesity classification and risk communication. Consequently, the World Health Organization in 1997 [15] and the US National Institutes of Health with 52 professional societies in 1998 [16] adopted BMI and waist circumference guidelines to support public understanding, prevention strategies, and patient-level interventions [15,16].

Soon after guideline adoption, studies using four-compartment models and dual-energy X-ray absorptiometry (DXA) in more diverse populations identified significant differences in BMI–body fat relationships by race, nationality, and age [17]. These findings were confirmed in early 2000s studies of Asian populations, which reported greater percent body fat (BF%) at lower BMI values than Caucasian populations [18,19]. Differences in direct measures of body fatness were also observed among Hispanic Americans, European Americans, and African Americans by 2003 [20], while European populations remained consistent with original BMI classifications [21]. Additionally, BMI-based obesity was not associated with increased mortality risk in African American women, despite associations in white American women [22].

Due to these limitations, including inadequacy for athletes, direct measures of body fatness have been promoted as health indicators since 2001 [3]. Measures of metabolic health have also been viewed as more informative than BMI for assessing health risk [22]. In direct comparisons, BF% predicts mortality risk more strongly than BMI in young adults [23]. Grip strength has likewise been associated with cardiorespiratory and cardiovascular mortality [24,25,26,27], particularly in men [28], as well as cause-specific and all-cause mortality in middle-aged [24,29] and older populations [29,30,31], especially older women [31].

Recent studies have reported significantly lower grip strength in BMI-defined obesity among South Korean women over 65 years [32]. Similar findings were also observed in Finnish men and women over 55 years, where reduced grip strength was strongly correlated with years lived with obesity [33]. Large studies across European and Asian populations further demonstrate that grip strength improves prediction of all-cause mortality in adults [34,35,36,37,38,39]. In contrast, BMI–mortality relationships are non-linear, and a major study found no increased mortality risk among BMI-defined overweight or mildly obese adults [7]. Gale et al. [7] suggested that muscle function, measurable via grip strength, may be a more important determinant of survival than body or muscle mass. Supporting this, Linge et al. [40] showed poor muscle function, characterized by low muscle volume and high fat infiltration, independently predicted all-cause mortality.

Given evidence linking grip strength to muscle function and volume, it is unsurprising that multiple measures of grip strength have been inversely associated with BF% in young adults across diverse populations [41,42,43,44,45]. Considering limitations of BMI for predicting BF%, particularly among athletes and non-European populations [3,17,18,19], and the importance of prolonged adiposity and muscle function for disease and mortality risk, we sought to determine whether combining grip strength measures with BMI improves prediction of BF% and obesity in a university population. As a secondary aim, we document relative grip strength, BMI, and BF% in a predominantly young adult population for enabling future studies that examine trends impacted by changes in exercise, labor practices, diet, and Glucagon-like peptide-1 (GLP-1) pharmaceuticals.

## 2. Materials and Methods

The study analyzed existing data from 895 individuals with complete data obtained from their participation in a fitness testing event, the Fitness Five Assessment, performed in the 2015–2016 academic year. The assessment was conducted at a regional university in Kentucky (USA), and included assessments of muscular strength, and body composition. For purposes of this study, data were limited to age, sex, BMI, BF%, and grip strength.

Participants were volunteers recruited from the general student population through a university-required wellness course at the university. Students who participated in the Fitness Five Assessment completed all necessary components of the assessment during a single assessment in 2015–2016 school year. All participants were 18 years of age or above. Opportunities to participate were available for two days during both the fall and spring semester. In accordance with an approved protocol by the Institutional Review Board of the university, all volunteers signed informed consent forms. Participants were made aware that their participation would not affect the outcome of their grade in any class or their status with the university. All participants completed the assessments in the same order. All assessments were performed by individuals trained to collect the data. Grip strength was collected for each hand using a hand grip dynamometer, recorded in pounds, and converted to kilograms (kg). Body composition analysis included a body fat analysis (BF%) using a handheld Bioelectrical Impedance Analysis and BMI.

### 2.1. BMI and BMI Categorizations

Both height (m) and weight (kg) information were collected via self-report and entered by the researcher into a hand-held bio-electrical impedance analysis machine. From that information, their BMI (kg/m^2^) was calculated. For enabling data analysis, the BMI classification for each participant was determined using the ranges set by the Expert Panel on the Identification, Treatment of Overweight, Obesity in Adults of the NIH [15]. Specifically, BMI (kg/m^2^) values were classified as underweight (<18.5), normal (18.5–24.9), overweight (25.0–29.9), or obese (BMI ≥ 30). Obesity classifications were further divided into class I (30.0–34.9), class II (35.0–39.9), and class III (≥40).

### 2.2. Hand Grip Dynamometer Strength Measurements

Hand grip strength measurements (grip strength) were obtained using a JAMAR Hydraulic Hand Dynamometer (Model J00105, Lafayette Instrument Company, Lafayette, IN, USA). The instrument was used with trained research personnel that supervised and collected grip strength measurements, specifically isometric grip force and strength, in pounds. For recording results, the peak-hold needle was used to retain the highest measurement for the left and right hand for each participant, except when participants had only one functional hand. Research personnel instructed and monitored participants to ensure their upper arm was at their side, their elbow was held at a 90-degree angle, and their thumb and fingertips touched, but did not overlap. Participants were advised to hold the device away from them like firing a pistol, and to squeeze the instrument for three to five seconds while keeping their arm and elbow steady. The measure obtained for each hand was converted to kg. Grip-to-BMI ratio (GBR), known as relative grip strength, was also obtained. Like other studies [41,42,43], GBR was calculated as the grip strength from the strongest hand (kg) divided by BMI to normalize muscle strength for body size.

No known cutoff points for sarcopenia are available for college-aged populations. For enabling comparison with other studies assessing sarcopenia risk and relatively strong grip strength values, cut points for sarcopenia risk among elderly men (<32 kg) and women (<23 kg) were used in the categorical data analysis [44] due to the lack of established cut points among young adults. Additionally, to enable comparison of relatively strong grip strength values, the upper cut point of 57 kg was used. This value corresponds approximately with the values deemed by one instrument supplier as strong for men in the age group of 20 to 29 years [45].

### 2.3. Body Fat Measurements and Sex-Based Body Fat Classifications

The BF% was determined for each participant using a hand-held Omron Fat Loss Monitor (Model HBF-306, Omron Healthcare, Kyoto, Japan), which has been used to obtain BF% in similar studies also incorporating grip strength [41,43,46]. Participants were instructed to self-report their height, weight, and age to the researcher. That information was recorded and then entered into the hand-held Omron Fat Loss Monitor. The researcher then instructed the participant to grasp the handles of the device and grip them firmly. They were told to hold the device out in front of their body with elbows extended and arms perpendicular to the body. The start button was pressed, and the subject held the device for 10 s while the instrument estimated their body composition under the supervision of the researcher. The instrument uses bioelectrical impedance analysis (BIA) methodology by sending a very low-level electrical current of 50 kHz and 500 µA to determine the amount of fat tissue upon adjusting for total body mass. The instrument provides the BF% as body fat mass divided by the body weight, then multiplied by 100. The body fat mass, and ultimately the BF%, were reported by the instrument upon providing inputs of height, body weight, age, and sex prior to the instrument obtaining an electric resistance measurement. The instrument manual indicates that body fat mass and BF% were “calculated by a formula that includes five factors: electric resistance, height, weight, age, and gender.” The manual does not provide the proprietary formula used for calculating or estimating BF%.

For enabling data analysis, obesity (excess body fat) as determined by BF% was defined as ≥25% for males (men) and ≥32% for females (women). While the obesity thresholds are not clearly established in the literature and may vary by age, the BF% was determined upon reviewing the literature for thresholds for BF% deemed obese or unhealthy. The 1998 joint position statement addressing obesity prevention, diagnosis, and treatment by the American Association of Clinical Endocrinologists (AACE) and the American College of Endocrinology (ACE) set obesity thresholds at 25% and 35% for men and women, respectively [47]. For recent studies, the obesity or unhealthy threshold for women, including young healthy adults, has been set at 32%, with the threshold being consistent for men at 25% [48,49], which aligns with the Lohman categorization [50]. These obesity thresholds (≥25% and ≥32%) are also used by the American Council on Exercise for men and women [51].

### 2.4. Statistical Analysis

The central aim of the analysis was to explore associations between grip strength, relative grip strength, and BF% for assessing the predictive potential of grip strength measures for BF% prediction and BF%-based obesity prediction. All statistical analyses were performed using Stata 15 (StataCorp, College Station, TX, USA) with the dataset available in Supplementary Materials (Table S1). Descriptive statistics were obtained for continuous variables such as BMI, BF%, age, dominant hand grip strength, etc. Descriptive analyses, notably frequency analyses, were performed on categorical variables such as ordinal BMI categories and BF% categories. Measures of central tendency were obtained for continuous variables. Analyses were performed by men and women subpopulations as well as the overall combined population. Pearson correlation and linear regression analyses were performed by sex with BF% as the dependent (outcome) variable. Independent (predictor) variables evaluated included BMI, age, grip strength, and relative grip strength. Multivariable linear regression analysis was performed to assess the strength of the association by each predictor in models and for assessing their relative importance for explaining the variability in the outcome (BF%) versus other independent variables. Following linear regression analyses and visual inspections of the model fits, Breusch–Pagan/Cook–Weisberg tests for heteroskedasticity were performed in Stata 15 using the “estat hettest” command. For linear models with heteroskedasticity (violating model assumptions), model terms and the overall model were re-evaluated for statistical significance using robust standard errors by adding the “robust” command after the regression commands. For examining collinearity, variance inflation factors were assessed using the “vif” command.

Additional analyses were performed using a binary classification of the outcome of interest (BF%), whereby men and women were classified as “BF_obese” (coded as 1) if their measured BF% was ≥25% and ≥32%, respectively. Persons classified as not obese by the BF% thresholds were coded as 0 for BF_obese. Similarly to a BMI-based approach, men and women were classified as “BMI_obese” (coded as 1) if their calculated BMI was ≥30. Persons with BMI values under 30 were coded as 0 for BMI_obese. A chi-square analysis compared the prevalence of obesity definitions by the BF% and BMI approaches, whereby *p* < 0.05 was set to indicate evidence of a significant difference. Chi-square analyses were also performed to evaluate if model-based estimates of BF% resulted in a significant difference in BF% obesity between model-predicted values and observed values.

Logistic regression was performed to assess predictors of BF%-based obesity (BF_obese) using the “logistic” command in Stata 15. Logistic models were specific to men and women. Models with one covariate (e.g., BMI) obtained crude odds ratios. Adjusted odds ratios were obtained for multivariable models including more than one covariate (e.g., BMI and grip strength). For visualizing marginal predicted probabilities of obesity as defined by BF%, the “marginsplot” command was used in conjunction with the “margins” command that enabled visualization of the men- and women-based logistic models at specific BMI and grip strength values. The margins used for BMI were set from 18.5 to 40 to illustrate model predictions (with 95% confidence intervals) for BF_obese across a range of BMI values from underweight through class III obesity. The margins used for dominant hand grip strength were the 25th, 50th (median), and 75th percentile measures in kg for men and women in their respective models.

Following the model runs, models were evaluated for discrimination (sensitivity and specificity), calibration, and simplicity. Continuous covariates were evaluated using the fractional polynomial method to assess linearity in the logit using the “fracpoly logit” command with the ‘compare’ option to obtain Likelihood Ratio Chi-Square test results. Specifically, for discrimination analysis, the area under the receiver–operator characteristic curve (area under the ROC curve) was obtained using the “lroc” command. Additional discrimination statistics, including sensitivity, specificity, predictive values, etc., were obtained using the “lstat” command. Model calibration was evaluated using the Hosmer–Lemeshow goodness-of-fit test using the “lfit, group (10) table” command, whereby the model expected frequencies of BF_obese were compared to the observed frequencies of BF_obese across 10 deciles of risk via the chi-square test. Lastly, Akaike’s information criterion (AIC) was obtained using the “estat ic” command for assessing and comparing model simplicity across the logit models.

## 3. Results

### 3.1. Summary Statistics

Complete data were available for 895 participants among 952 participants who provided grip strength data as part of the Fitness Five Assessment. Among the 952 participants, there were missing data related to body weight (*n* = 52), height (*n* = 32), and age (*n* = 14). Overall, 401 (45%) study participants with complete data were women and 494 (55%) were men. As participants were participating while enroled in a lower division university course, freshmen and sophomores were more represented than the overall general student body. Accordingly, the average ages of the men and women in the study were 19.8 and 20.1 years with the median age for both men and women being 19 years old (Table 1). The median BMI values for both men (24.8) and women (23.4) were within the normal BMI range; however, the mean BMI of 25.8 for men categorizes as overweight. Overall, the frequency of obesity as determined by adult BMI was 17% in men and 12.7% among women (Table 2). Table 2 demonstrates that the frequency of BF% below 20% was greater for men than women (68% versus 23%, respectively).

Table 2 demonstrates clear differences between men and women in the distributions of BMI, body fat, grip strength, and GBR. Men in this study were more frequently classified as overweight or obese (49.2% combined) relative to women (32.7%). Alternatively, when examining body fat differences, 24.9% of women had BF% values exceeding 30%, relative to 7.1% of the men in the study. In terms of grip strength and GBR, men had considerably greater grip strength and GBR values. Table 1 and Table 2 show that half of the men in the study had grip strength and GBR values of 47.6 kg and 1.9, respectively; these values are considerably greater than the median values of 31.8 kg and 1.3 among the women participants.

### 3.2. Obesity Prevalence by BMI- and Body Fat-Based Obesity Definitions

Among the 895 participants with complete data, there was no difference in the frequency of obesity determined by the BF% method versus the BMI method (*p* = 0.302). In men, the frequency of obesity was very similar, with 16.6% of men having body fat-based obesity versus 17.0% having BMI-based obesity (Table 3). For women, there was more disagreement between the body fat-based obesity prevalence and the BMI-based obesity prevalence with 17.2% and 12.7%, respectively. As the *p*-value approached a significant difference (*p* = 0.075), the results suggest the BMI-based approach under-classified obesity (relative to the body fat-based method) when examining the results in aggregate by sex.

### 3.3. Linear Regression Analyses for Predicting Body Fat Percentages

**Figure 1:**
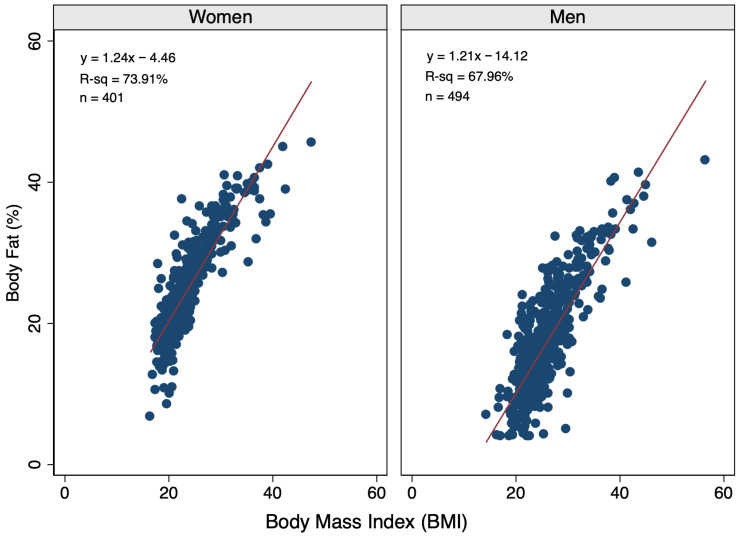
Scatterplots by sex illustrating the relationship between body mass index and body fat percentage in the study population with linear regression lines and equations included.

**Figure 2:**
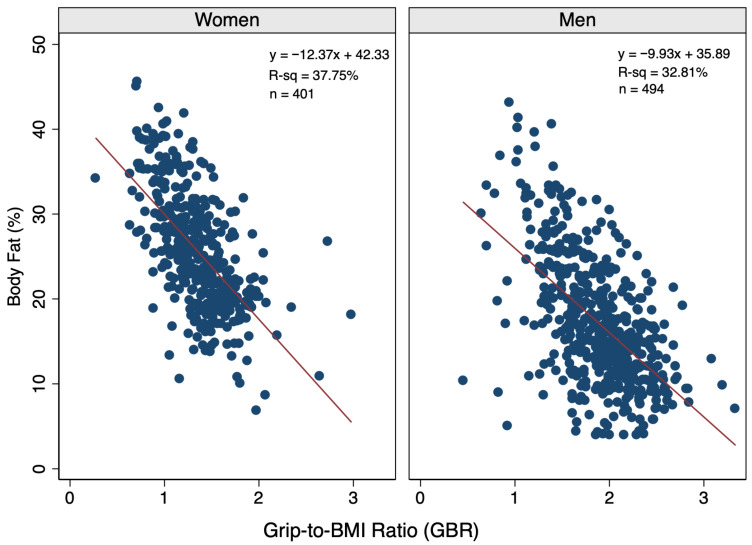
Scatterplots by sex illustrating the relationship between the grip-to-BMI ratio (GBR) and the body fat percentage in the study population with linear regression lines and equations included, whereby the GBR represents the dominant hand grip strength (kg) divided by the BMI.

BMI and GBR were significant predictors (*p* < 0.0001) of BF% in sex-based linear regression models informed by 401 women and 494 men. In simple (one-predictor) models, dominant hand grip strength (kg) was not a significant predictor of BF% for women (R^2^ = 0.0003, *p* = 0.729) or men (R^2^ = 0.0011, *p* = 0.454). When comparing BMI versus GBR, BMI was a stronger predictor of BF% for both sexes. Specifically, BMI explained 73.91% and 67.96% of the variability in BF% among women and men, respectively (Figure 1), whereas GBR explained 37.75% and 32.25% of BMI variability (Figure 2). Because BMI and GBR were moderately correlated among women (r = 0.532) and men (r = 0.545), sex-specific multivariable models incorporated hand grip strength (kg) rather than GBR (kg/BMI) to reduce multicollinearity in women (VIF = 1.02 vs. 1.39) and men (VIF = 1.05 vs. 1.42), and because GBR and BMI are not independent terms, as GBR contains BMI in its denominator. Specifically, grip strength has greater independence of BMI than GBR, and grip strength–BMI correlations were lower (r = 0.208 women; r = 0.143 men).

**Figure 3:**
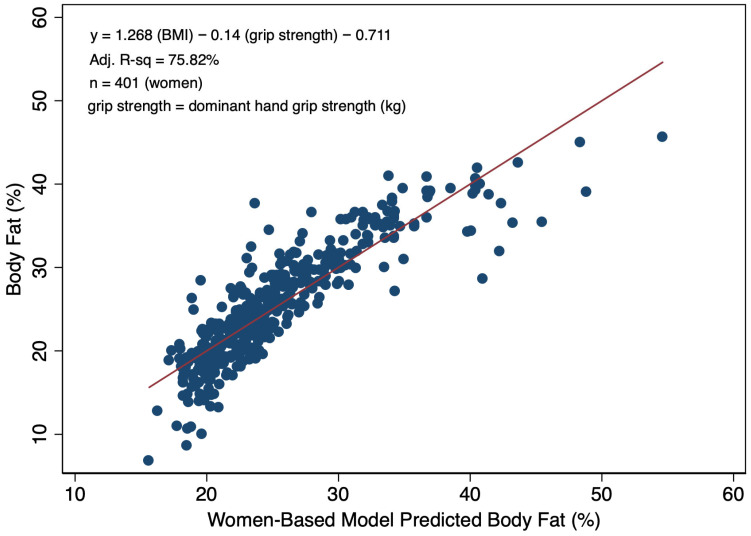
Scatterplot with a line-of-best-fit illustrating the relationship between BIA-determined body fat percentage and model-predicted body fat percentages informed by BMI and dominant hand grip strength (kg) for adult women.

**Figure 4:**
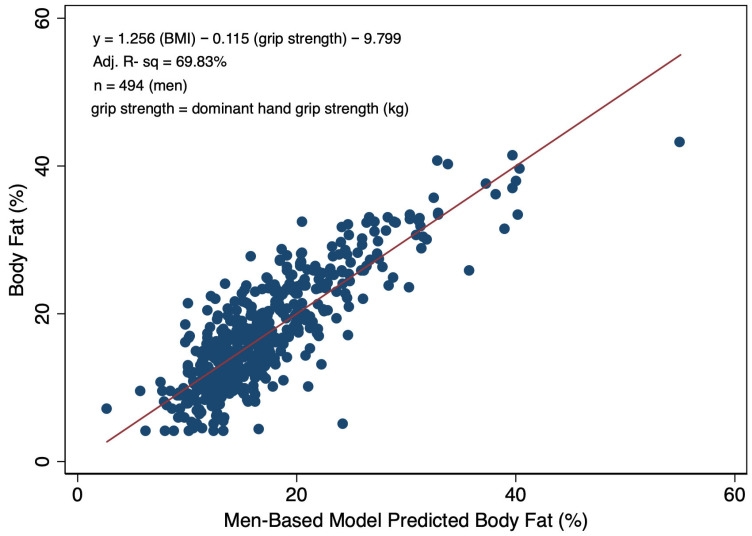
Scatterplot with a line-of-best-fit illustrating the relationship between BIA-determined body fat percentage and model-predicted body fat percentages informed by BMI and dominant hand grip strength (kg) for adult men.

While dominant hand grip strength was not a significant predictor of BF% alone, in multivariable linear models among women and men predicting BF% and including BMI, the grip strength term was significant. Specifically, for every one kg increase in grip strength, there was a 0.140% increase in BF% among women (Figure 3). Among men, each one kg increase in grip strength was associated with a 0.115% decrease BF% (Figure 4). The inclusion of the grip strength measure along with BMI presented a modest increase in model precision in both the women- and men-based models versus using BMI alone. Specifically, the model including BMI and grip strength explained 76% of the variability in BF% among women versus 74% in men. Similarly, a 2% increase (70% vs. 68%) was also observed in the percent of the variability explained in the model specific to men when grip strength was incorporated.

For developing a single multivariable linear equation for predicting BF%, a model was developed that accounted for sex. The inclusion of the sex term increased the percent of variability in BF% explained by the model to 79.25% (Figure S1). In that model, the BF% for men was, on average, 8.5% lower than the BF% for women. Like the models specific to men and women, grip strength had an inverse relationship with BF%, whereby each one kg increase in grip strength was associated with a 0.12% decrease in BF% upon adjusting for BMI and sex.

Upon defining obesity as BF% over 32% and 25% for women and men, respectively, cross-tabulations illustrate that, at these cut points for obesity, the BMI-based determinations of non-obese individuals have strong agreement, as evidenced by specificity values of 97.9% for women and 94.6% for men (Table 4). When incorporating grip strength, the specificity values improve to 98.5% for women and 99.0% for men (Table 5). However, sensitivity (the ability to detect body fat-determined obesity) is substantially lower in both women and men. The BMI-based method correctly classifies body fat-based obesity in 63.7% of women and 75.6% of men (Table 4). Incorporating grip strength enhanced sensitivity in women (73.9%), but decreased sensitivity in men (52.4%). In evaluating these models using the scatterplots (Figure 4 and Figure S1), the linear fitted line demonstrated statistically significant heteroscedasticity (Chi-Square *p* < 0.05) with larger variances being observed at the higher predicted values, suggesting potential to improve diagnostic abilities using nonlinear approaches. While heteroscedasticity was present in models, the models (F-test *p* < 0.0001) and model terms (*t*-test *p* < 0.001) remained statistically significant when robust standard errors were incorporated.

### 3.4. Logistic Regression Analyses for Predicting Body Fat-Based Obesity

Simple and multivariable logistic regression models for predicting obesity by body fat (BF_obese) were developed for men and women with the following covariates: BMI, grip strength, and age. In evaluating logistic regression models, age was not linear in the logit (Chi-Square *p* = 0.017); however, BMI (*p* = 0.161) and grip strength (*p* = 0.161) were not different from linearity. Accordingly, age was dichotomized to over 19 years old or not. For women, the binary age covariate was not significant (aOR = 1.65, 95% C.I.: 0.62–4.38; *p* = 0.313) in multivariable models and it had no significant impact on the other covariates. For men, the binary age covariate was significant and is displayed in the M3 model (Table 6). Specifically, the adjusted odds ratios (aOR) in the M3 model demonstrate that young men (age 18 or 19 years of age) had a 279% or 3.79-times greater likelihood of having more than 25% body fat than the men in the study who were 20 years of age or older upon adjusting for BMI and grip strength (aOR = 3.79, 95% CI: 1.61–8.96, *p* = 0.002).

In examining the five logit models (F1, F2, M1, M2, and M3), increasing BMI levels were linked to an increased likelihood of having body fat-based obesity in every model. In the multivariable models for women (F2) and men (M3), for every one unit increase in BMI, the odds of having body fat-based obesity increased 100% (2×) and 97% (1.97×), respectively, upon adjusting for grip strength. Grip strength had a protective effect in the same models, whereby upon adjusting for BMI, each one kg increase in grip strength was associated with a 10% and 6% decrease in the likelihood of body fat-based obesity in women and men, respectively (Table 6).

**Figure 5:**
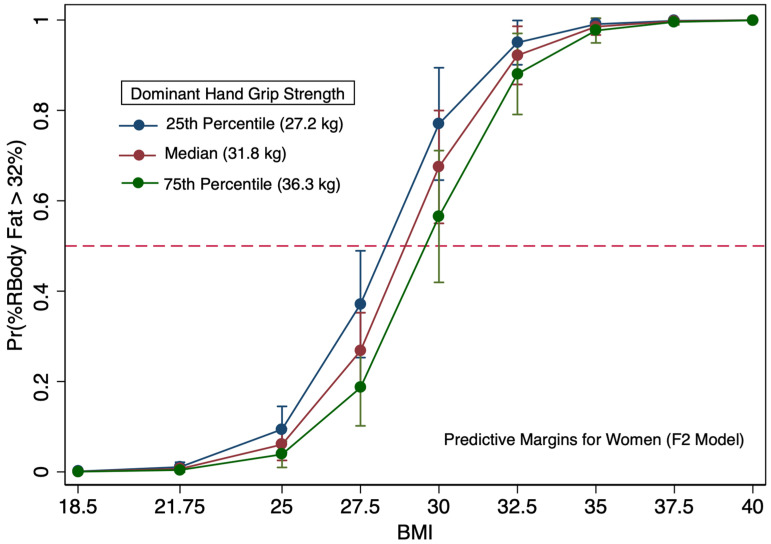
Predicted probabilities of elevated body fat (≥32%) by BMI (kg/m^2^) among women with predictive margins displayed by the dominant hand grip strength percentiles (25th, 50th, and 75th) of 401 women.

The predicted probabilities of body fat-based obesity (BF_obese) are depicted for women (F2 model) and men (M2 model) accounting for BMI and grip strength and no age covariate in Figure 5 and Figure S2. Overall, grip strength has some influence on the likelihood of meeting body fat-based obesity criteria when the BMI is near 30 (27.5–32.5) in female and male models. The impact of grip strength on reducing the likelihood of BF–based obesity is negligible when BMI scores are near 35. For women in this study with a BMI of 32.5 at the 75th percentile of grip strength (36.3 kg), the likelihood of body fat-based obesity (BF% > 32%) was near 80%. Alternatively, women with overweight BMI values (27.5 BMI) having the lowest quartile of grip strength (27.2 kg) had a probability of less than 40% of being obese.

In the M2 model, which does not account for age, grip strength in men, like in women, appears to have very little to no influence on the probability of being classified as obese by the BF%-based approach when BMI values are near 35, whereby predicted probabilities exceed 90% across all grip strength levels (Figure S2). For men with a BMI at 32.5 and with the 75th percentile of grip strength for men (54.4 kg), their likelihood of having over 25% body fat (BF_obese) is approximately two-thirds. Unlike the women with a BMI of 30, most men with a BMI of 30 would not be expected to be obese by the body fat method in this study. Among men, even those with a relatively low grip strength (at the 25th percentile [40.8 kg]) were more likely than not to have a BF% below 25%.

Interestingly, when ascertaining what amount of dominant hand grip strength is associated with having body fat-based obesity in women (≥32%) and men (≥25%) with a BMI of 30, the value is the same, at 38.8 kg. For women in the study, a grip strength of 38.8 kg was in the top quartile, whereas 38.8 kg was in the bottom quartile among men.

**Figure 6:**
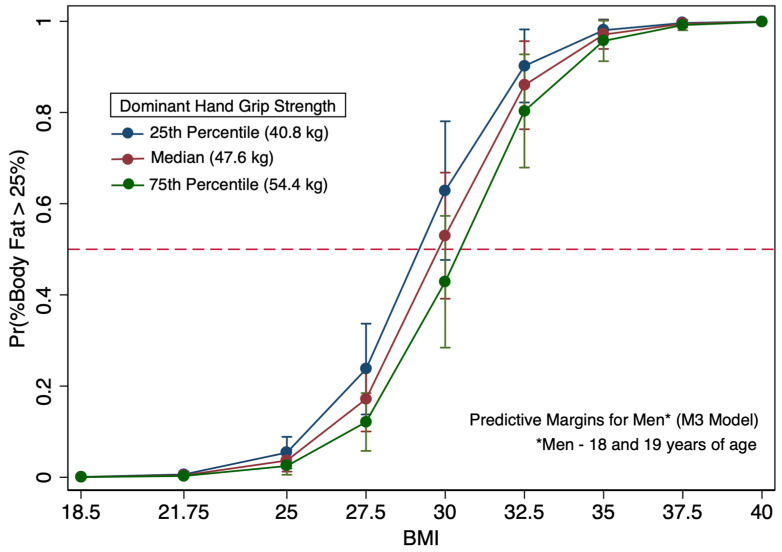
Predicted probabilities of elevated body fat (≥25%) by BMI (kg/m^2^) for men of 18 or 19 years of age with predictive margins displayed by the dominant hand grip strength percentiles (25th, 50th, and 75th) of 494 men.

**Figure 7:**
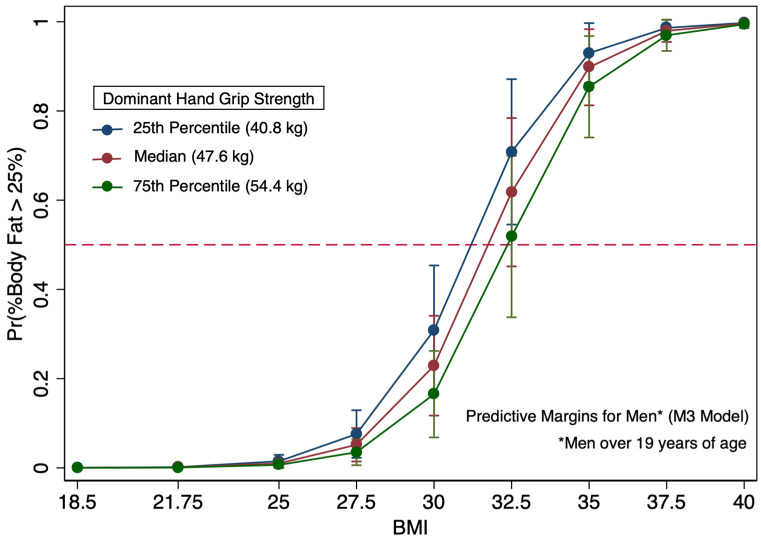
Predicted probabilities of elevated body fat (≥25%) by BMI (kg/m^2^) for men over 19 years of age with predictive margins displayed by the dominant hand grip strength percentiles (25th, 50th, and 75th) of 494 men.

It is important to note that logistic regression models specific to men showed evidence of confounding by age, as illustrated by the changes in grip strength and BMI coefficients between the M2 and M3 models upon addition of the age category covariate to the M3 model (Table 6). The impact is apparent when comparing Figure 6 and Figure 7. In Figure 6, which is specific to the youngest men in the study (age 18 and 19), among those with a BMI of 30, most had more than 25% body fat. For young men (age 18 and 19) with a BMI of 30, a grip strength of 49.6 kg, which is above the median grip strength for all the men in the study, was needed to have a 50% probability of being at the body fat-based obesity threshold. Alternatively, men over 19 years of age in the M3 model with a BMI of 30 needed a 27.3 kg grip strength to be at 50% probability of body fat-based obesity (Figure 7). In this study population, the BMI and BF%-based categorizations of obesity are in greater alignment among women and the younger men. Men over 19 years of age with BMI values of 30 typically had a BF% that would not constitute obesity by a BF% method.

An evaluation of diagnostic performance for the five logistic regression models showed all models demonstrated outstanding discrimination, with areas under the ROC curve exceeding 90% [52]. All the models provided acceptable calibration, whereby the model-expected results were not significantly different (goodness-of-fit *p* > 0.05) than the observed results across 10 deciles of risk for body fat-based obesity. Overall, the F2 and M3 models performed best among the women- and men-based models, as evidenced by the highest area under ROC curve values and lowest AIC values (Table 7).

## 4. Discussion

Although DXA and bioelectrical impedance analysis (BIA) provide more detailed assessments of muscle quality and body composition, their strong correlation with grip strength in children and young adults suggests that grip strength may serve as a practical proxy or screening tool [53]. For reasons similar to why BMI was adopted, notably practicality, a grip strength-based approach could provide utility in clinical or resource-limited settings where prognostic health information is gathered and used for health education, interventions, or screening efforts determining a need for referral to advanced methods. Unlike BMI, grip strength has demonstrated consistency in estimating lean muscle mass by DXA across different cultures, albeit among older study populations [54]. Future studies are needed to see how the measures perform across different races and ethnicities in younger populations. Given that BMI (or BMI as a sole predictor) is inadequate for distinguishing adiposity, prior research encourages the inclusion of muscular fitness measures, such as handgrip strength, coupled with BMI to accurately predict BF% in adults and children [55,56]. A substantial and growing body of evidence is now linking functional measures, specifically low grip strength, as a marker of sarcopenia and metabolic disorders in children, teenagers, and young adults, which have apparent impacts on their healthspans and quality of life [57,58,59,60,61]. Recently, thresholds or cut points indicative of metabolic disease risks have been developed using normalized grip strength for consideration in children and young adults [58,61]. Based upon these recent findings, it is beneficial to reflect upon our study of a mostly young adult population in the context of potential uses of hand grip strength measures for informing health interventions and public education.

### 4.1. Grip Strength Possibly Inadequate as a Sole Predictor of Adiposity for Young Adults

Attempts to develop guidelines may be informed by the existing literature examining hand grip strength versus levels of adiposity deemed excessive or other measures used for defining metabolic disease. In deriving normative reference values for adult men and women 18 to 85 years of age in the US, grip strength values were significantly impacted by the age, height, and weight of participants [62]. In studies focusing on children and young adults, normalization of grip strength was done to adjust for body mass [56,58,61]. In our study, the grip-to-BMI ratio, also known as relative handgrip strength, was significantly correlated with the likelihood of body fat-based obesity; however, grip strength alone (kg) was not linked to BF% until paired with a measure of body size. While the findings we present are focused on estimating BF%, it is likely that a body mass adjustment (either mass or BMI) for grip strength would be needed in other diagnostic or surveillance endeavors aimed at linking grip strength to health outcomes of interest. Given that BF% was determined by the instrument using a prediction algorithm which utilizes body height and weight, it is plausible that BMI was an artificially strong predictor of BF% in our models. It is plausible that instrument equation which estimates BF% rather than truly measures BF% attenuated the potential effect of grip strength alone. Given that the BF% prediction equation of the manufacturer uses height and weight, the grip strength variable may have been strengthened when paired with BMI due to a lack of independence between the dependent variable (BF%) and the independent variables.

In examining the logistic regression models only using BMI for men (M1) and women (F1), both models had areas under the ROC curve exceeding 95% for model discrimination indicating outstanding discrimination as a diagnostic tool for predicting body fat-based obesity. Other research using more diverse populations and that accounted for the covariates age, sex, and ethnicity found that all three covariates impacted their models predicting body fat. Our study, which was conducted at a predominantly white institution, had insufficient data from non-white participants, and we could not assess ethnicity impacts on BMI. Our study population also demonstrated less age diversity than many other BMI-oriented BF% studies. Studies with more diverse populations observed BMI not being an excellent indicator of obesity; however, our results demonstrated BMI as an outstanding indicator of excess body fat in a predominantly white population. These findings are not new and confirm research that illustrates BMI can predict BF% in many persons, particularly white European populations [16,18]. Despite having outstanding discrimination, since 2008, BMI-based approaches have been known to have problems with sensitivity for detecting excessive BF% among persons deemed overweight or mildly obese by BMI, resulting in misclassification [63]. Table 7 demonstrates that the grip strength measure included in the F2 model increased sensitivity by approximately 10% in women; however, it did not increase overall sensitivity in the models specific to men. By examining the marginal plots, persons with a BMI in the overweight to mildly obese range have substantially different likelihoods of being classified as having excessive body fat, depending upon their grip strength (Figure 5, Figure 6 and Figure 7).

While there are limitations to the generalizability of the data, the fact that grip strength made a modest statistically significant improvement in the BF% estimate warrants further research, particularly due to the greater likelihood that proprietary models which used BMI attenuated some of the grip strength effect in this university population.

### 4.2. Age and Sex Implications for Health-Relevant Grip Strength Criteria

Like many other studies [28,31,53,62,63], we observed that grip strength, particularly relative grip strength, varies significantly by sex and age. Noticeable in our study and DeHondt et al. [61], a significant increase in normalized or relative grip strength was observed among young men as they aged. We observed this phenomenon as considerable differences between the population of young men aged 18 and 19 years versus the men over 19 years in age. The similarity of relative grip strength demonstrated in our university population relative to other studies is described in Supplementary Text S1.

Overall, the relative grip strength values and grip strength measures from this study are within the range of previous research on young adults [61]. Based upon our findings and previously cited research, adiposity-based criteria using grip strength may be difficult to establish for young adults undergoing development, particularly young men, even when coupled with BMI. Age categories for young adults, particularly young men, may need to be smaller than 18–24 years of age, as the age covariate is likely not linear in logit models aiming to predict excess body fat. For grip strength, men aged 18 to 19 with an obese BMI of 30 would need to have a grip strength of at least 49.6 kg to have an estimated BF% below 25%, whereas a young man in his 20s also with a BMI of 30 could have a substantially weaker grip strength (27.3 kg or greater) to have a BF% below 25%. Future studies using grip strength that focus on adiposity and related health indictors or outcomes likely need to consider the significance of confounding presented by adult development extending to 24 years, especially young men [64]. This effect of a considerable gain in grip strength near the age of 19 in men is likely generalizable to other populations.

### 4.3. Implausibly Low Body Fat Percentage Estimates and BIA Instrument Limitations

The results of this study should be treated cautiously, as the outcome variable (BF%) generated abnormal results. Specifically, the range of BF% measures in this study included an instrument-generated BF% level of less than 4.5% in nine men, and less than 6% in 17 men. Additionally, the instrument produced a value of 6.8% and 8.6% in two women. While performance bodybuilders can achieve the levels observed in these men prior to competitions, most of these levels are not observed in athletes who have been evaluated by superior methods like DXA [65]. If using the 10% and 16% minimum thresholds for essential body fat in male athletes and female athletes [66], 16% of the men and 5% of the women in our study had BIA-reported BF% values below these thresholds, demonstrating measurement errors for BF%. The reasons for these measurement errors involving the BIA instrument may include study participants misreporting needed inputs (age, weight, height), and/or user errors while holding the instrument not observed by the supervising researcher. While BIA, including the Omron instrument, has been strongly correlated with gold standard BF% determination methods such as DXA and densitometry [20], more recent research, particularly with younger populations, have observed Omron instrument measures of BF% being significantly different from DXA, with an average BF% difference as high as 3.6% to 4.9% [65,66].

In addition to the potential errors we observed, the instrument manufacturer indicates time since last meal, hydration status, recent exercise, and/or fatigue may impact results. While BIA results are correlated with BF%, the strength of the correlation with the true BF% for this device is unknown, so there is the potential that the BF% prediction algorithm is inaccurate for unique individuals. Presumably, these types of errors could exist for all the participants, which could make misclassifications of body fat-based obesity non-differential, and could thus enable the directional assumptions about the associations regarding grip strength and BF% to hold true at the population level for men and women.

Another challenge with the BIA-based method used was the device measures hand-to-hand or arm-to-arm. The BF% estimates obtained from the instrument may not be true measures of overall BF%, as newer commercially available BIA instruments assess foot-to-foot as well as foot-to-hand and have different modes (with presumably different BF% equations) specific to user-entered activity or fitness level (e.g., athlete mode). Future studies likely would benefit from incorporating physical activity levels. Given this study focused on hand grip strength, it may be acceptable to attempt to correlate these measures, since both are upper body measures. Future research on grip strength relative to other measures of whole-body composition versus upper body composition could be considered. Additionally, consecutive measures of grip strength, measures of hand grip endurance, or related measures such as timed free hanging may have enhanced prediction of BF% and may also enhance prediction of lean muscle mass. These types of research on practical strength measures could be enhanced by examining potentially more health-relevant outcomes beyond BF%, such as ones afforded by reference methods such as DXA inclusive of indices for lean mass, fat-free mass, and/or appendicular lean mass.

In contrast to our study, a 2023 study by Doğan et al. [67] of young adults (18–30 years) was able to explore the relationship between grip strength and body fatness utilizing anthropometric variables (e.g., waist circumference, skinfold thickness, etc.), thereby reducing the inherent challenges of BIA instruments that use proprietary algorithms which include BMI, creating a lack of independence between outcome and predictor variables. From their study, they observed waist circumference may offer greater clinical relevance, and their findings suggest waist circumference would be predictive of hand grip strength. Therefore, future studies examining this relationship of grip strength and waist circumference may hold promise in health prediction models or other healthspan-oriented body composition studies.

### 4.4. Assessment of Generalizability to University Populations

As the wellness course was a required course at the time of study, it was more representative of the university community than discipline-specific courses. However, the population in a lower division course was disproportionately first-year (freshman) and second-year (sophomore) students, and thus younger than the university or national university populations, which would have more third-year (juniors) and fourth-year (seniors) students if drawn at random. Given the predominantly white population, the results may not be generalizable to a more demographically-diverse US college population. Furthermore, the university services a low-income region of the US, whereby slightly more than 50% of students are eligible for federal (US) Pell grants [68], which are offered to students with federally-defined exceptional financial need.

Since Pell students are more likely to be food insecure relative to students not using Pell aid [69, 70], these students may be more food insecure than university students elsewhere in the US and beyond. Furthermore, these students may be more likely to be characterized as obese by the BMI method relative to students who do not experience food insecurity [71]. For comparing with national data, the men in this study had 17% obesity relative to 13% in the national US assessment of college students the same year of this study [72]. Women in this study, however, had lower self-report informed obesity (12.7%) than the national assessment of college women (14.9%) [72]. Despite having a population at greater risk for obesity, we did not observe considerable differences from national data; however, self-reported body weight may have been greater than actual among men and lower among women, possibly due to a gender-specific social desirability bias [73].

### 4.5. Study Limitations

The findings presented have significant limitations regarding their generalizability and clinical relevance. The study intent was to explore the potential of grip strength for estimating excessive body fat or contributing to evaluations of body fatness. For making greater generalizations, the study has several important limitations that warrant considerable caution in the interpretations and illustrate areas for research improvements when incorporating BIA devices into research. Specifically, this study has a major statistical limitation related to independence or the likely lack thereof between the outcome (BF%) and the predictors used (age, sex, and BMI). While the instrument manual does not directly require the user to enter BMI, the manual indicates the formula uses height and weight, which are the basis of the BMI calculation. Other studies acknowledge that BIA instruments use proprietary formulas [72]. Therefore, we cannot conclude that the very strong relationship between BF% and BMI is true, as it may be artificially inflated because the BF% is not a true measure of BF% but rather the instrument-based estimate informed by the same variables used to calculate BMI. It is plausible that independent measures like grip strength were attenuated in our models, and future studies using raw impedance measures be explored if developing statistical models as age and sex are also not independent in our models. Specifically, age and sex also were factored into the BF% calculation by the instrument and were also among the predictor variables. Future studies using BIA for body fat analysis must remain aware of the risk of a lack of independence in their BF% outcome (dependent) and predictor (independent) variables if BF% is provided by these types of instruments rather than resistance (R).

Beyond the major limitation of statistical independence, we have additional limitations related to (1) a high risk of measurement errors from self-reported height and weight, (2) the cross-sectional study design, (3) the convenience sampling approach potentially not representative of the university population, (4) the lack of information on race/ethnicity of participants, (5) the body fat-based thresholds for obesity, (6) the lack of alternative measures of metabolic and/or general health, and (7) the lack of data regarding the participants’ lifestyles such as the absence of information on physical activity levels. The manual for the handheld BIA instrument used in this study describes challenges in obtaining reliable results in several populations, including bodybuilders, athletes, patients with very low bone density, pregnant women, and others.

Beyond the measurement errors, the generalizability of the study was impacted by lack of a truly random sample from the general student population. Specifically, the convenience sampling approach used increased the potential for participation biases with outgoing or health-conscientious students being overrepresented relative to the general student population. Additional challenges with the cross-sectional nature of the study is that a single measure of BF% may not be an accurate long-term reflection of grip strength or vice versa, and repeated measure designs offer greater insights. Lastly, the population studied reflected the diversity of the university and the Commonwealth of Kentucky, USA. Race and ethnicity were not evaluated in this study, and the institution is approximately 85% white. Numerous studies related to anthropometric measures and human health have observed race and/or ethnicity variables to be useful [17,18,19,20,21,22], and they continue to be recommended for inclusion in future research.

## 5. Conclusions

BMI was a strong predictor of body fat for most individuals in this predominantly white, American, university-based young adult population. However, despite its overall predictive strength, there was evidence that BMI lacks sensitivity for identifying individuals with excessive body fat relative to sex-specific thresholds. When combined with handgrip strength, sensitivity improved notably among women. Among young men, grip strength also enhanced sensitivity, though to a lesser extent than observed in women. Importantly, model-based predictions of excess body fat in young men were strongly influenced by developmental differences, suggesting that future studies should exercise caution when grouping men aged 18 to 24 years together to establish health thresholds for body fat or grip strength. Differences between men under and over 19 years of age were particularly pronounced. Overall, this study adds to growing evidence that muscle function—not body mass alone—contributes meaningfully to predicting health-relevant outcomes such as body fat percentage. Incorporating functional measures alongside traditional anthropometric indicators may improve diagnostic classification, support more effective screening and health education, and better target individuals most at risk, until advanced diagnostic methods become more accessible.

## Figures and Tables

**Table 1:** Descriptive statistics for anthropometric measures, grip strength, and age among study participants summarized by sex.

Covariate	Sex	Mean	SD	Median	Range
BMI	Women	24.3	4.8	23.4	16.5–47.4
BMI	Men	25.8	5.1	24.8	14.3–56.5
BMI	Combined	25.1	5.0	24.1	14.3–56.5
Body Fat (%)	Women	25.6	6.9	25.2	6.8–45.6
Body Fat (%)	Men	17.2	7.5	15.9	4.0–43.1
Body Fat (%)	Combined	21.0	8.4	20.4	4.0–45.6
Dominant Grip (kg)	Women	32.0	7.1	31.8	9.1–72.6
Dominant Grip (kg)	Men	47.5	9.4	47.6	8.4–79.4
Dominant Grip (kg)	Combined	40.5	11.4	38.6	8.4–79.4
Grip-to-BMI Ratio (GBR)	Women	1.4	0.3	1.3	0.3–3.0
Grip-to-BMI Ratio (GBR)	Men	1.9	0.4	1.9	0.5–3.3
Grip-to-BMI Ratio (GBR)	Combined	1.6	0.5	1.6	0.3–3.3
Age (years)	Women	19.8	4.2	19	18–53
Age (years)	Men	20.1	4.0	19	18–59
Age (years)	Combined	19.9	4.1	19	18–59

**Table 2:** Distribution of anthropometric and grip strength results and age among study participants summarized for women (*n* = 401) and men (*n* = 494).

Adult BMI Category	Women	Men
Underweight (<18.5 kg/m^2^)	17 (4.2%)	8 (1.6%)
Normal (18.5–24.9 kg/m^2^)	253 (63.1%)	243 (49.2%)
Overweight (25.0–29.9 kg/m^2^)	80 (20.0%)	159 (32.2%)
Obese Class I (30.0–34.9 kg/m^2^)	33 (8.2%)	56 (11.3%)
Obese Class II & III (≥35.0 kg/m^2^)	18 (4.5%)	28 (5.7%)
**Body Fat Category**		
Below 20%	92 (22.9%)	335 (67.8%)
20% to 24.99%	105 (22.9%)	77 (15.6%)
25% to 29.99%	104 (25.9%)	47 (9.5%)
30% to 34.99%	51 (12.7%)	25 (5.1%)
35% or Greater	49 (12.2%)	10 (2.0%)
**Dominant Hand Grip Strength**		
Below 23.0 kg	29 (7.2%)	5 (1.0%)
23.0–32.0 kg	205 (51.0%)	22 (4.4%)
32.1 to 36.0 kg	58 (14.4%)	19 (3.8)
36.1 to 43.0 kg	88 (21.9%)	118 (23.7%)
43.1. to 50.0 kg	17 (4.2%)	158 (31.7%)
50.1 kg or 57.0 kg	2 (0.5%)	112 (22.4%)
57.1 kg or Greater	3 (0.7%)	65 (13.0%)
**Grip-to-BMI Ratio (GBR)**		
GBR < 1.0	57 (14.2%)	12 (2.4%)
GBR 1.0–1.49	222 (55.4%)	83 (16.8%)
GBR 1.5–1.99	112 (27.9%)	199 (40.2%)
GBR 2.0 or Greater	10 (2.5%)	200 (40.5%)
**Age Category**		
18 to 19 (years)	293 (73.3%)	313 (63.4%)
20 to 24 (years)	93 (23.3%)	158 (32.8%)
25 to 59 (years)	14 (3.5%)	23 (4.7%)

**Table 3:** A comparison of obesity prevalence using two obesity determination methods stratified by sex.

Sex	Obese by Body Fat Method ^1^	Obese by BMI Method ^2^	Chi-Sq *p*
Women	69/401 (17.2%)	51/401 (12.7%)	0.075
Men	82/494 (16.6%)	84/494 (17.0%)	0.865
Combined	151/895 (16.9%)	135/895 (15.1%)	0.302

^1^ Body fat cut points for obesity were ≥32% and 25% in women and men, respectively. ^2^ The BMI cut points for obesity were ≥30 for both women and men.

**Table 4:** Cross-tabulations demonstrating the frequency of agreement of obesity classifications made by the BMI versus classifications determined through sex-specific body fat percentages.

Classification Group by Body Fat %	Classification by BMI (Obese ≥ 30 kg/m^2^)		Classification Agreement	
	Not Obese	Obese	*n*/N (%)	Statistic Type
Women—Not Obese	325	7	325/332 (97.9%)	Specificity
Women—Obese (>32%)	25	44	44/69 (63.7%	Sensitivity
Men—Not Obese	390	22	390/412 (94.6%)	Specificity
Men—Obese (>25%)	20	62	62/82 (75.6%)	Sensitivity
Total—Not Obese	715	29	715/744 (96.1%)	Specificity
Total—Obese	45	106	106/151 (70.2%)	Sensitivity

**Table 5:** Cross-tabulations demonstrating the frequency of agreement in obesity classifications made by sex-specific body fat percentages determined by BIA versus multivariable linear regression model estimates for body fat percentage determined by BMI, dominant hand grip strength (kg), and sex.

Classification Group by Body Fat %	Linear Model Body Fat Classification		Classification Agreement	
	Not Obese	Obese	*n*/N (%)	Statistic Type
Women—Not Obese	327	5	327/332 (98.5%)	Specificity
Women—Obese (>32%)	18	51	51/69 (73.9%)	Sensitivity
Men—Not Obese	408	4	408/412 (99.0%)	Specificity
Men—Obese (>25%)	39	43	43/82 (52.4%)	Sensitivity

**Table 6:** Simple (one covariate) and multivariable (two or more covariates) logistic regression models for predicting an obese body fat classification for women (≥32%) and men (≥25%) along with corresponding crude and adjusted odds ratios (OR) for covariates, dominant hand grip strength (kg), and biological sex.

Model	Covariate	b	S.E. _b_^1^	*p*	OR ^2^	OR 95% CI
Women F1	BMI (kg/m^2^)	0.639	0.075	<0.001	1.89	1.63–2.20
	Constant	−18.561	2.084			
Women F2	BMI (kg/m^2^)	0.694	0.083	<0.001	2.00	1.70–2.36
	Grip Strength (kg)	−0.104	0.035	0.003	0.90	0.84–0.97
	Constant	−16.795	2.154			
Men M1	BMI (kg/m^2^)	0.592	0.065	<0.001	1.81	1.59–2.05
	Constant	−18.342	1.900			
Men M2	BMI	0.623	0.068	<0.001	1.86	1.63–2.12
	Grip Strength (kg)	−0.056	0.021	0.007	0.95	0.91–0.98
	Constant	−16.507	1.959			
Men M3	BMI (kg/m^2^)	0.678	0.076	<0.001	1.97	1.69–2.29
	Grip Strength (kg)	−0.060	0.021	0.004	0.94	0.90–0.98
	Age 18 or 19 (years)	−1.333	0.439	0.002	3.79	1.61–8.96
	Constant	−18.697	2.233			

^1^ SE _b_: Standard error (SE) of the model coeffficents (b). ^2^ OR: Odds Ratio (OR) presented as a crude odds ratio for one covariate models (F1 and M1) and as an adjusted odds ratio for models with more than one covariate (F2, M2, and M3).

**Table 7:** Summary of model diagnostics for simple (F1, M1) and multivariable (F2, M2, M3) logistic regression models aimed at predicting an obese body fat classification for adult men and women.

Model Diagnostic	Model				
	F1	F2	M1	M2	M3
Area Under ROC Curve (%)	95.51	96.47	95.47	95.99	96.66
Sensitivity (%)	72.46	81.16	68.29	68.29	70.73
Specificity (%)	97.59	98.19	98.06	96.84	97.33
Positive Predictive Value (%)	86.21	90.32	87.5	81.16	84.06
Correct Classification (%)	93.27	95.26	93.12	92.11	92.91
Goodness-of-Fit *p*	0.597	0.243	0.789	0.778	0.421
Akaike Information Criterion	156.98	148.96	198.53	192.62	184.25

## Data Availability

The original data presented in the study are openly available as Supplementary Material.

## Supplementary Materials

The following supporting information can be downloaded at https://www.mdpi.com/article/10.3390/diagnostics16081210/s1; Table S1: A spreadsheet file (.xlsx) containing the data for 901 study participants organized across 21 variables (columns) on sheet 1 (DataSheet); sheet 2 (CodeSheet) describes the variable (column) names. Figure S1: Scatterplot with a line-of-best-fit illustrating the relationship between BIA-determined body fat percentage and model-predicted body fat percentages informed by BMI, sex (man or woman), and dominant hand grip strength (kg). Figure S2: Predicted probabilities of elevated body fat (≥25%) by BMI (kg/m^2^) among men with predictive margins displayed by the dominant hand grip strength percentiles (25th, 50th, and 75th) of 494 men. Supplemental Text S1: Comparison of relative grip strength values obtained relative to other studies.

## Abbreviations

The following abbreviations are used in this manuscript:

BF%	Body Fat Percentage
AACE	American Association of Clinical Endocrinologists
ACE	American College of Endocrinology
AIC	Akaike information criterion
aOR	Adjusted odds ratio
AUC	Area under the receiver operating characteristic curve
BF	Body fat
BF_obese	Body fat-based obesity classification
BIA	Bioelectrical impedance analysis
BMI	Body mass index
CI	Confidence interval
DXA	Dual-energy X-ray absorptiometry
GBR	Grip-to-BMI ratio
NIH	National Institutes of Health
OR	Odds ratio
R^2^	Coefficient of determination
ROC	Receiver operating characteristic
SD	Standard deviation
WHO	World Health Organization

## References

[1] Ng M., Dai X., Cogen R.M., Abdelmasseh M., Abdollahi A., Abdullahi A., Aboagye R.G., Abukhadijah H.J., Adeyeoluwa T.E., Afolabi A.A. (2024). National-level and state-level prevalence of overweight and obesity among children, adolescents, and adults in the USA, 1990–2021, and forecasts up to 2050. Lancet.

[2] Speakman J.R., Sørensen T.I., Hall K.D., Allison D.B. (2023). Unanswered questions about the causes of obesity. Science.

[3] Prentice A.M., Jebb S.A. (2001). Beyond body mass index. Obes. Rev..

[4] Keys A., Fidanza F., Karvonen M.J., Kimura N., Taylor H.L. (1972). Indices of relative weight and obesity. J. Chronic Dis..

[5] Eknoyan G. (2008). Adolphe Quetelet (1796–1874)—The average man and indices of obesity. Nephrol. Dial. Transplant..

[6] Pray R., Riskin S., Riskin S.I. (2023). The history and faults of the body mass index and where to look next: A literature review. Cureus.

[7] Nuttall F.Q. (2015). Body mass index: Obesity, BMI, and health: A critical review. Nutr. Today.

[8] Stamler J. (1993). Epidemic obesity in the United States. Arch. Intern. Med..

[9] Manson J.E., VanItallie T.B. (1996). America’s obesity epidemic and women’s health. J. Womens Health.

[10] Egger G., Swinburn B. (1997). An “ecological” approach to the obesity pandemic. BMJ.

[11] Kannel W.B., Cupples L.A., Ramaswami R., Stokes J., Kreger B.E., Higgins M. (1991). Regional obesity and risk of cardiovascular disease: The Framingham Study. J. Clin. Epidemiol..

[12] Manson J.E., Colditz G.A., Stampfer M.J., Willett W.C., Rosner B., Monson R.R., Speizer F.E., Hennekens C.H. (1990). A prospective study of obesity and risk of coronary heart disease in women. N. Engl. J. Med..

[13] Ford E.S., Williamson D.F., Liu S. (1997). Weight change and diabetes incidence: Findings from a national cohort of US adults. Am. J. Epidemiol..

[14] World Health Organization (2000). Obesity: Preventing and Managing the Global Epidemic.

[15] Expert Panel on the Identification, Evaluation, and Treatment of Overweight and Obesity in Adults (US), National Heart, Lung, and Blood Institute, National Institute of Diabetes and Digestive and Kidney Diseases (US) (1998). Clinical Guidelines on the Identification, Evaluation, and Treatment of Overweight and Obesity in Adults: The Evidence Report.

[16] Gallagher D., Heymsfield S.B., Heo M., Jebb S.A., Murgatroyd P.R., Sakamoto Y. (2000). Healthy percentage body fat ranges: An approach for developing guidelines based on body mass index. Am. J. Clin. Nutr..

[17] Tan K.C.B. (2004). Appropriate body-mass index for Asian populations and its implications for policy and intervention strategies. Lancet.

[18] Deurenberg P., Deurenberg-Yap M., Guricci S. (2002). Asians are different from Caucasians and from each other in their body mass index/body fat per cent relationship. Obes. Rev..

[19] Fernández J.R., Heo M., Heymsfield S.B., Pierson R.N., Pi-Sunyer F.X., Wang Z.M., Wang J., Hayes M., Allison D.B., Gallagher D. (2003). Is percentage body fat differentially related to body mass index in Hispanic Americans, African Americans, and European Americans?. Am. J. Clin. Nutr..

[20] Deurenberg P., Andreoli A., Borg P., Kukkonen-Harjula K., De Lorenzo A., van Marken Lichtenbelt W.D., Testolin G., Viganò R., Vollaard N. (2001). The validity of predicted body fat percentage from body mass index and from impedance in samples of five European populations. Eur. J. Clin. Nutr..

[21] Stevens J., Cai J., Jones D.W. (2002). The effect of decision rules on the choice of a body mass index cutoff for obesity: Examples from African American and white women. Am. J. Clin. Nutr..

[22] Lassale C., Tzoulaki I., Moons K.G., Sweeting M., Boer J., Johnson L., Huerta J.M., Agnoli C., Freisling H., Weiderpass E. (2018). Separate and combined associations of obesity and metabolic health with coronary heart disease: A pan-European case-cohort analysis. Eur. Heart J..

[23] Mainous A.G., Yin L., Wu V., Sharma P., Jenkins B.M., Saguil A.A., Nelson D.S., Orlando F.A. (2025). Body mass index vs body fat percentage as a predictor of mortality in adults aged 20–49 years. Ann. Fam. Med..

[24] Celis-Morales C.A., Lyall D.M., Anderson J., Iliodromiti S., Fan Y., Ntuk U.E., Mackay D.F., Pell J.P., Sattar N., Gill J.M. (2017). The association between physical activity and risk of mortality is modulated by grip strength and cardiorespiratory fitness: Evidence from 498 135 UK Biobank participants. Eur. Heart J..

[25] Mearns B.M. (2015). Hand grip strength predicts cardiovascular risk. Nat. Rev. Cardiol..

[26] Prasitsiriphon O., Pothisiri W. (2018). Associations of grip strength and change in grip strength with all-cause and cardiovascular mortality in a European older population. Clin. Med. Insights Cardiol..

[27] Chainani V., Shaharyar S., Dave K., Choksi V., Ravindranathan S., Hanno R., Jamal O., Abdo A., Abi Rafeh N. (2016). Objective measures of the frailty syndrome (hand grip strength and gait speed) and cardiovascular mortality: A systematic review. Int. J. Cardiol..

[28] Gale C.R., Martyn C.N., Cooper C., Sayer A.A. (2007). Grip strength, body composition, and mortality. Int. J. Epidemiol..

[29] Sasaki H., Kasagi F., Yamada M., Fujita S. (2007). Grip strength predicts cause-specific mortality in middle-aged and elderly persons. Am. J. Med..

[30] Bohannon R.W. (2019). Grip strength: An indispensable biomarker for older adults. Clin. Interv. Aging.

[31] Arvandi M., Strasser B., Meisinger C., Volaklis K., Gothe R.M., Siebert U., Ladwig K.H., Grill E., Horsch A., Laxy M. (2016). Sex differences in the association between grip strength and mortality in older adults: Results from the KORA-Age study. BMC Geriatr..

[32] Park K.N., Kim S.H. (2022). Comparison of grip strength, gait speed, and quality of life among obese, overweight, and nonobese older adults: A cross-sectional study. Top. Geriatr. Rehabil..

[33] Stenholm S., Sallinen J., Koster A., Rantanen T., Sainio P., Heliövaara M., Koskinen S. (2011). Association between obesity history and hand grip strength in older adults—Exploring the roles of inflammation and insulin resistance as mediating factors. J. Gerontol. A Biol. Sci. Med. Sci..

[34] Celis-Morales C.A., Welsh P., Lyall D.M., Steell L., Petermann F., Anderson J., Iliodromiti S., Sillars A., Graham N., Mackay D.F. (2018). Associations of grip strength with cardiovascular, respiratory, and cancer outcomes and all-cause mortality: Prospective cohort study of half a million UK Biobank participants. BMJ.

[35] Chai L., Zhang D., Fan J. (2024). Comparison of grip strength measurements for predicting all-cause mortality among adults aged 20+ years from the NHANES 2011–2014. Sci. Rep..

[36] López-Bueno R., Andersen L.L., Calatayud J., Casaña J., Grabovac I., Oberndorfer M., del Pozo Cruz B. (2022). Associations of handgrip strength with all-cause and cancer mortality in older adults: A prospective cohort study in 28 countries. Age Ageing.

[37] Wang Y., Liu Y., Hu J., Guan H., Wang Y., Liu M., He L., Sun N., Yang W., Ma Y. (2022). Association of handgrip strength with all-cause mortality: A nationally longitudinal cohort study in China. J. Sci. Med. Sport.

[38] Laukkanen J.A., Voutilainen A., Kurl S., Araujo C.G.S., Jae S.Y., Kunutsor S.K. (2020). Handgrip strength is inversely associated with fatal cardiovascular and all-cause mortality events. Ann. Med..

[39] Kim J. (2021). Handgrip strength to predict the risk of all-cause and premature mortality in Korean adults: A 10-year cohort study. Int. J. Environ. Res. Public Health.

[40] Linge J., Petersson M., Forsgren M.F., Sanyal A.J., Dahlqvist Leinhard O. (2021). Adverse muscle composition predicts all-cause mortality in the UK Biobank imaging study. J. Cachexia Sarcopenia Muscle.

[41] Pettersson-Pablo P., Nilsson T.K., Hurtig-Wennlöf A. (2024). Relative handgrip strength correlates inversely with increased body fat, inflammatory markers and increased serum lipids in young, healthy adults: The LBA study. Diabetes Res. Clin. Pract..

[42] Garcia-Hermoso A., Tordecilla-Sanders A., Correa-Bautista J.E., Peterson M.D., Izquierdo M., Prieto-Benavides D., Sandoval-Cuellar C., González-Ruíz K., Ramírez-Vélez R. (2019). Handgrip strength attenuates the adverse effects of overweight on cardiometabolic risk factors among collegiate students but not in individuals with higher fat levels. Sci. Rep..

[43] Abdelnour D., Grove M., Pulford-Thorpe K., Windhurst K., LeCrone C., Kerr E., Hew-Butler T. (2025). Associations between absolute and relative handgrip strength with fitness and fatness. Sports Med. Int. Open.

[44] Bahat G., Tufan A., Tufan F., Kilic C., Akpinar T.S., Kose M., Erten N., Karan M.A., Cruz-Jentoft A.J. (2016). Cut-off points to identify sarcopenia according to European Working Group on Sarcopenia in Older People (EWGSOP) definition. Clin. Nutr..

[45] Camry (2025). User Manual: EH101 Digital Hand Dynamometer Grip Strength Meter.

[46] Dhar D.P., Purwar B. (2023). Effect of body fat and BMI on muscle strength and endurance in young adults: A cross-sectional study. J. Clin. Diagn. Res..

[47] Dickey R.A., Bartuska D., Bray G.W., Callaway C.W., Davidson E.T., Feld S., Ferraro R.T., Hodgson S.F., Jellinger P.S., Kennedy F.P. (1998). AACE/ACE position statement on the prevention, diagnosis, and treatment of obesity (1998 revision). Endocr. Pract..

[48] Yogesh M., Mody M., Makwana N., Patel J. (2024). Exploring the silent epidemic: Investigating the hidden burden of normal weight obesity in type 2 diabetes mellitus in India—A cross-sectional study. Clin. Diabetes Endocrinol..

[49] Mondal H., Mishra S.P. (2017). Effect of BMI, body fat percentage and fat free mass on maximal oxygen consumption in healthy young adults. J. Clin. Diagn. Res..

[50] Lohman T.G. (1992). Advances in Body Composition Assessment: Current Issues in Exercise Science.

[51] Dorwart L. Body Fat Percentage: Charting Averages in Men and Women. American Council on Exercise.

[52] Hosmer D.W., Lemeshow S., Sturdivant R.X. (2013). Applied Logistic Regression.

[53] Richardson C.G., Opotowsky A.R., Chin C., Mays W.A., Knecht S.K., Powell A.W. (2025). The relationship of handgrip strength to body composition and cardiopulmonary fitness in children and young adults. J. Pediatr. Clin. Pract..

[54] Moncada-Jiménez J., Dicker E.E., Chacón-Araya Y., Peralta-Brenes M., Briceño-Torres J.M., Villarreal-Ángeles M., Salazar-Villanea M., Vidoni E.D., Burns J.M., Johnson D.K. (2023). Exploring handgrip strength as a cross-cultural correlate of body composition and upper body strength in older adults from Costa Rica and Kansas. J. Cross-Cult. Gerontol..

[55] Nickerson B.S., Esco M.R., Bishop P.A., Fedewa M.V., Snarr R.L., Kliszczewicz B.M., Park K.S. (2018). Validity of BMI-based body fat equations in men and women: A four-compartment model comparison. J. Strength Cond. Res..

[56] Steffl M., Chrudimsky J., Tufano J.J. (2017). Using relative handgrip strength to identify children at risk of sarcopenic obesity. PLoS ONE.

[57] Laitinen T.T., Saner C., Nuotio J., Sabin M.A., Fraser B.J., Harcourt B., Juonala M., Burgner D.P., Magnussen C.G. (2020). Lower grip strength in youth with obesity identifies those with increased cardiometabolic risk. Obes. Res. Clin. Pract..

[58] Ko D.H., Kim Y.K. (2021). The prevalence of metabolic syndrome according to grip strength in teenagers. Children.

[59] Cao M.Y., Yan W., Shi Y.N., Peng L.T., Zheng Q.Q., Gao S.H., Zhao M., Wang L., Li X.N. (2025). Relationship between skeletal muscle mass and strength with metabolic syndrome in children. Zhonghua Er Ke Za Zhi.

[60] Jung H.W., Lee J., Kim J. (2022). Handgrip strength is associated with metabolic syndrome and insulin resistance in children and adolescents: Analysis of Korea National Health and Nutrition Examination Survey 2014–2018. J. Obes. Metab. Syndr..

[61] DeHondt B.G., Madi S.A., Drignei D., Buchan D.S., Brown E.C. (2023). Handgrip strength cut-points for cardiometabolic risk identification in US younger population. Meas. Phys. Educ. Exerc. Sci..

[62] Wang Y.C., Bohannon R.W., Li X., Sindhu B., Kapellusch J. (2018). Hand-grip strength: Normative reference values and equations for individuals 18 to 85 years of age residing in the United States. J. Orthop. Sports Phys. Ther..

[63] Romero-Corral A., Somers V.K., Sierra-Johnson J., Thomas R.J., Collazo-Clavell M.L., Korinek J.E.C., Allison T.G., Batsis J.A., Sert-Kuniyoshi F.H., Lopez-Jimenez F. (2008). Accuracy of body mass index in diagnosing obesity in the adult general population. Int. J. Obes..

[64] Sawyer S.M., Azzopardi P.S., Wickremarathne D., Patton G.C. (2018). The age of adolescence. Lancet Child Adolesc. Health.

[65] Burns R.D., Fu Y., Constantino N. (2019). Measurement agreement in percent body fat estimates among laboratory and field assessments in college students: Use of equivalence testing. PLoS ONE.

[66] Olson K.L. (2019). Body Composition Methods. Master’s Thesis.

[67] Doğan G., Tokaç Er N., Öztürk M.E., Meriç Ç.S., Yilmaz H.Ö., Yabanci Ayhan N. (2025). Hand grip strength in young adults: Association with obesity-related anthropometric variables. J. Public Health.

[68] National Center for Education Statistics College Navigator. Eastern Kentucky Univeristy. https://nces.ed.gov/collegenavigator/?q=eastern+kentucky&s=all&id=156620#finaidA.

[69] Royer M.F., Ojinnaka C.O., Bruening M. (2021). Food insecurity is related to disordered eating behaviors among college students. J. Nutr. Educ. Behav..

[70] El Zein A., Colby S.E., Zhou W., Shelnutt K.P., Greene G.W., Horacek T.M., Olfert M.D., Mathews A.E. (2020). Food insecurity is associated with increased risk of obesity in US college students. Curr. Dev. Nutr..

[71] American College Health Association (2017). National College Health Assessment: Fall 2016 Reference Group Executive Summary. https://www.acha.org/wp-content/uploads/2024/07/NCHA-II_FALL_2016_REFERENCE_GROUP_EXECUTIVE_SUMMARY.pdf.

[72] Khan S., Xanthakos S.A., Hornung L., Arce-Clachar C., Siegel R., Kalkwarf H.J. (2020). Relative accuracy of bioelectrical impedance analysis for assessing body composition in children with severe obesity. J. Pediatr. Gastroenterol. Nutr..

[73] Quick V., Byrd-Bredbenner C., Shoff S., White A.A., Lohse B., Horacek T., Kattelmann K., Phillips B., Hoerr S.L., Greene G. (2015). Concordance of self-report and measured height and weight of college students. J. Nutr. Educ. Behav..

