# Supplementary material for: Combined Measure of Hand Grip Strength and Body Mass Index for Predicting Excess Body Fat in a University Population in Kentucky, USA
Source: Diagnostics (Basel). 2026 Apr 17;16(8):1210. doi: 10.3390/diagnostics16081210 (PMC13114614; doi:10.3390/diagnostics16081210)
Supplement: Supplementary file 1 [file diagnostics-16-01210-s001.zip › Suppl_FigureS1.pdf]

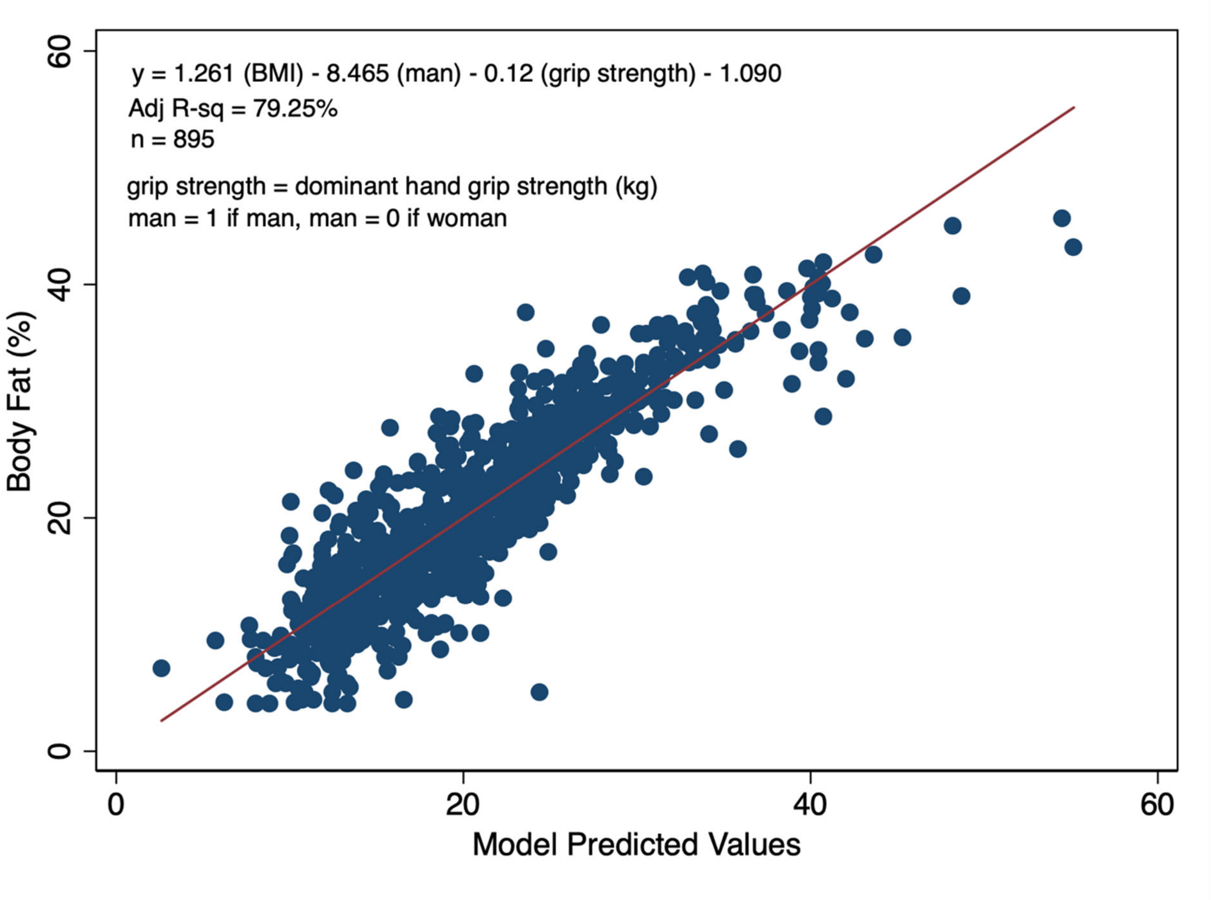

Figure S1. Scatterplot with a line-of-best-fit illustrating the relationship between BIA-determined body fat percentage and model-predicted body fat percentages informed by BMI, sex (man or woman), and dominant hand grip strength (kg).
