# Supplementary material for: Combined Measure of Hand Grip Strength and Body Mass Index for Predicting Excess Body Fat in a University Population in Kentucky, USA
Source: Diagnostics (Basel). 2026 Apr 17;16(8):1210. doi: 10.3390/diagnostics16081210 (PMC13114614; doi:10.3390/diagnostics16081210)
Supplement: Supplementary file 1 [file diagnostics-16-01210-s001.zip › Suppl_FigureS2.pdf]

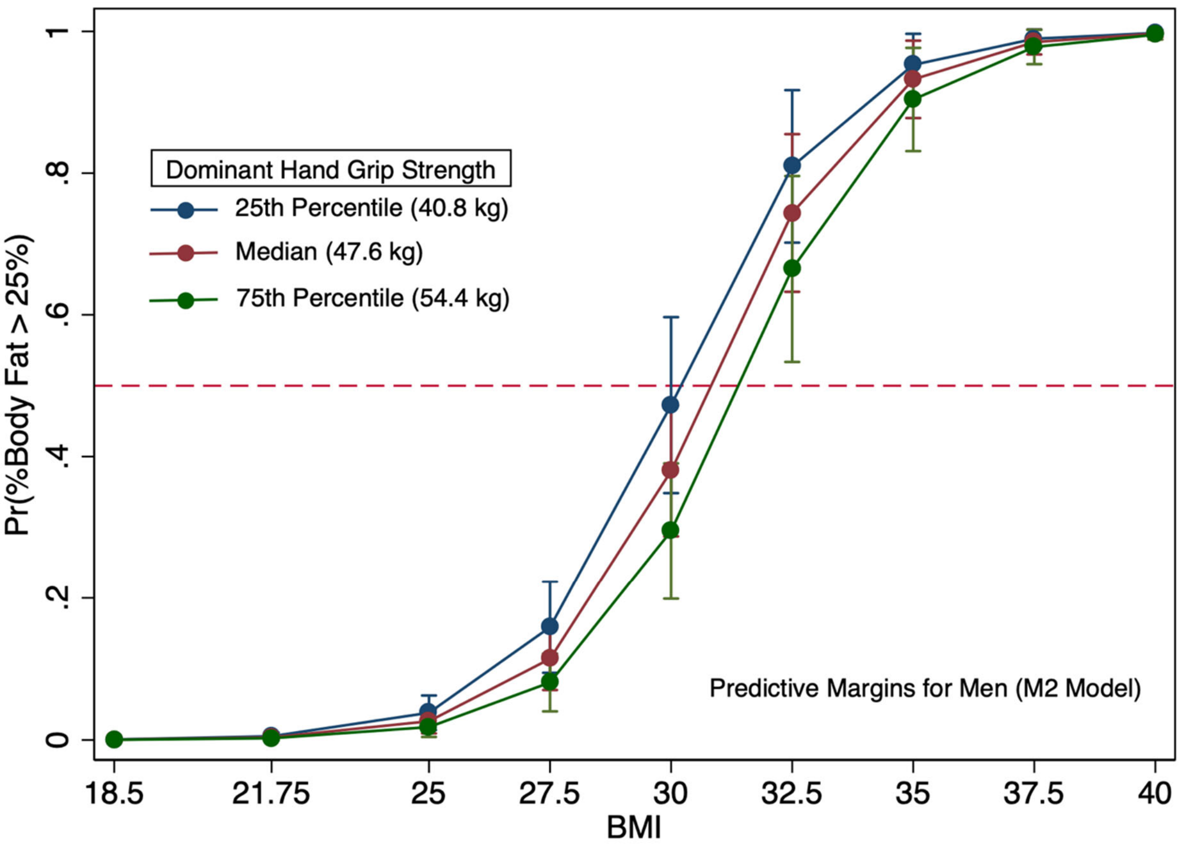

Figure S2. Predicted probabilities of elevated body fat (≥ 25%) by BMI (kg/m^2) among men with predictive margins displayed by the dominant hand grip strength percentiles (25th, 50th, and 75th) of 494 men.
