# Supplementary material for: Combined Measure of Hand Grip Strength and Body Mass Index for Predicting Excess Body Fat in a University Population in Kentucky, USA
Source: Diagnostics (Basel). 2026 Apr 17;16(8):1210. doi: 10.3390/diagnostics16081210 (PMC13114614; doi:10.3390/diagnostics16081210)
Supplement: Supplementary file 1 [file diagnostics-16-01210-s001.zip › Supplemental Text.pdf]

Supplemental Text

Comparison of Relative Grip Strength Values Obtained Relative to Other Studies

Similar to many other studies [1 - 5], we observed that grip strength, particularly relative grip strength, varies significantly by sex and age. In a study of US individuals by Wang et al. [4], among 18 – 24-year-olds, mean grip strength values were 28.1 kg and 47.0 kg for women (n = 36) and men (n = 54), respectively. In our study, women and men had slightly higher grip strength values at 32.0 kg and 47.5 kg, respectively, but also reflect a university population rather than a more generalized US population. Also, in Wang et al. [4], reference equations exist to estimate grip strength for men and women incorporating age, height, and weight terms. Based upon their equations, we estimated mean grip strength using the average age, height, and body weight for the women and men in this study. Using their equation for women assuming mean age of 19.8 years, 1.6 m for height, and 65.9 kg for body weight, we would expect a grip strength of 30.3 kg which is closer to our 32.0 kg average than the mean values in their 18 – 24-year-old group. Similarly, for men assuming mean age of 20.1 years, 1.8 m for height, and 83.5 kg for body weight, we would expect a grip strength of 48.8 kg, which also is closer to our value of 47.5 kg than their average from their 18 – 24-year-old group.

In a study by DeHondt et al. [6], normalized grip strength (grip-kg / body mass-kg) was examined in two age groups: 12 – 17 years and 18 – 24-years. For comparison, the mean body mass for women and men in our study was 65.9 kg and 83.5 kg, respectively, yielding normalized grip strength values of 0.43 for women and 0.56 for men. In DeHondt et al., among their 18 – 24-year-old group, they observed slightly higher values of 0.46 and 0.61 for men and women, respectively. When comparing our results to the younger group in DeHondt et al. (ages 12 – 17 years), we have more parity; whereby they observed 0.47 for women and 0.56 for men. The mean age in years of our study population was 19.8 and 20.1 among the women and men. Noticeable in these studies is the significant improvement in normalized or relative grip strength among young men. We observed this phenomenon as we observed considerable differences between the population of young men aged 18 and 19 years versus the men over 19 years in age.

1. Gale, C.R.; Martyn, C.N.; Cooper, C.; Sayer, A.A. Grip strength, body composition, and mortality. Int. J. Epidemiol. 2007, 36, 228–235.
2. Arvandi, M.; Strasser, B.; Meisinger, C.; Volaklis, K.; Gothe, R.M.; Siebert, U.; Ladwig, K.H.; Grill, E.; Horsch, A.; Laxy, M.; et al. Sex differences in the association between grip strength and mortality in older adults: results from the KORA-Age study. BMC Geriatr. 2016, 16, 201.
3. Richardson, C.G.; Opotowsky, A.R.; Chin, C.; Mays, W.A.; Knecht, S.K.; Powell, A.W. The relationship of handgrip strength to body composition and cardiopulmonary fitness in children and young adults. J. Pediatr. Clin. Pract. 2025, 16, 200144.
4. Wang, Y.C.; Bohannon, R.W.; Li, X.; Sindhu, B.; Kapellusch, J. Hand-grip strength: normative reference values and equations for individuals 18 to 85 years of age residing in the United States. J. Orthop. Sports Phys. Ther. 2018, 48, 685–693.
5. Romero-Corral, A.; Somers, V.K.; Sierra-Johnson, J.; Thomas, R.J.; Collazo-Clavell, M.L.; Korinek, J.E.C.; Allison, T.G.; Batsis, J.A.; Sert-Kuniyoshi, F.H.; Lopez-Jimenez, F. Accuracy of body mass index in diagnosing obesity in the adult general population. Int. J. Obes. 2008, 32, 959–966.
6. DeHondt, B.G.; Madi, S.A.; Drignei, D.; Buchan, D.S.; Brown, E.C. Handgrip strength cut-points for cardiometabolic risk identification in US younger population. Meas. Phys. Educ. Exerc. Sci. 2023, 27, 224–233.
